# Anticholinergic syndrome in a patient with schizophrenia

**DOI:** 10.1192/j.eurpsy.2022.2041

**Published:** 2022-09-01

**Authors:** O. Marco Estrada, L. Tardon, T. Fernández, L. Navarro, M. Bioque, N. Arbelo

**Affiliations:** 1 Hospital Clínic de Barcelona, Psychiatry, Barcelona, Spain; 2 Barcelona Clinic Schizophrenia Unit (BCSU), Neuroscience Institute, Hospital Clinic Of Barcelona, University Of Barcelona, Idibaps, Cibersambarcelona Clinic Schizophrenia Unit (bcsu), Neuroscience Institute, Barcelona, Spain; 3 Hospital Clínic Barcelona, Psychiatry, Barcelona, Spain

**Keywords:** anticholinergic, physostigmine, schizophrénia, Antipsychotics

## Abstract

**Introduction:**

Anticholinergic syndrome (AS) is a complication that can appear due to different drugs with antimuscarinic effects, such as antihistamines, alkaloids, antipsychotics, tricyclic antidepressives or anesthetics, and it is characterized by urinary retention, dry mouth and skin, mydriasis, low-grade fever, and confusion or coma.

**Objectives:**

To describe a clinical case of AS admitted to our hospital.

**Methods:**

We present a case report of a patient with schizophrenia who presented an anticholinergic syndrome. We also searched for previous studies of AS using a pubmed query.

**Results:**

A 53-year-old male was admitted for a psychotic decompensation to another hospital in Barcelona. The usual treatment at home was amisulpride 1200mg/d, olanzapine 30mg/d and lormetazepam, and haloperidol 6mg/d and clotiapine 40mg/d were added to treat the decompensation. Then, the patient started to present mydriasis, mucocutaneous dryness, low-grade fever, slight hypertension and tachycardia, repeated retentions of urine, confusion, unintelligible speech and agitation, so he was referred to our hospital. Once he was admitted, haloperidol was withdrawn and support measures (bladder catheterization, fluid therapy, etc.) were applied. After a few days, most of the mentioned alterations were stabilized, but the psychotic symptoms, such as thought and behavioural disorganization, persisted and required electroconvulsive therapy, with subsequent improvement.

**Conclusions:**

AS is a relatively frequent side effect of psychiatric medication, which diagnosis is clinical, so, we must be capable to identify it and initiate early treatment to prevent possible complications. The first step, as reflected in the case described, is to stop the causative drugs, and apply support measures. Additionally, physostigmine can be used, as it is an effective antidote.

**Disclosure:**

No significant relationships.

